# Foraging for carotenoids: do colorful male hihi target carotenoid-rich foods in the wild?

**DOI:** 10.1093/beheco/aru076

**Published:** 2014-05-12

**Authors:** Leila K. Walker, Rose Thorogood, Filiz Karadas, David Raubenheimer, Rebecca M. Kilner, John G. Ewen

**Affiliations:** ^a^Department of Zoology, University of Cambridge, Downing Street, Cambridge CB2 3EJ, UK,; ^b^Institute of Zoology, Zoological Society of London, Regent’s Park, London NW1 4RY, UK,; ^c^Department of Animal Science, Faculty of Agriculture, University of Yüzüncü Yil, Van 35080, Turkey, and; ^d^Charles Perkins Centre and Faculty of Veterinary Science and School of Biological Sciences, University of Sydney, Sydney, NSW 2006, Australia

**Keywords:** carotenoids, foraging, hihi, nutrition, plumage.

## Abstract

Dietary access to carotenoids is expected to determine the strength of carotenoid-based signal expression and potentially to maintain signal honesty. Species that display carotenoid-based yellow, orange, or red plumage are therefore expected to forage selectively for carotenoid-rich foods when they are depositing these pigments during molt, but whether they actually do so is unknown. We set out to address this in the hihi (*Notiomystis cincta*), a New Zealand passerine where males, but not females, display yellow carotenoid-based plumage. We measured circulating carotenoid concentrations in male and female hihi during breeding and molt, determined the nutritional content of common foods in the hihi diet, and conducted feeding observations of male and female hihi during molt. We found that although male and female hihi do not differ significantly in plasma carotenoid concentration, male hihi have a greater proportion of carotenoid-rich foods in their diet than do females. This is a consequence of a greater fruit and lower invertebrate intake than females and an avoidance of low-carotenoid content fruit. By combining behavioral observations with quantification of circulating carotenoids, we present evidence that colorful birds forage to maximize carotenoid intake, a conclusion we would not have drawn had we examined plasma carotenoids alone.

## Lay Summary

Birds that color their feathers with dietary carotenoid pigments are expected to seek out these pigments when they are molting. We show that molting male hihi, who express carotenoid-based plumage, seek out naturally occurring foods that are rich in carotenoid pigments. Female hihi, who do not express carotenoid-based plumage, do not seek out carotenoid-rich foods. This lends strength to the idea that carotenoid-based plumage reveals an individual’s foraging ability.

## INTRODUCTION

The brightly colorful, showy plumage displayed by males of many bird species has always fascinated biologists, forming the cornerstone of research on sexual selection and contributing to the development of theory on honest signals (Andersson 1994; Hill and McGraw 2004). Despite knowing how these colors are formed, and why they may function as signals, we still do not know if birds actively seek to be at their most colorful.

The key to understanding this lies in how birds access pigments. Reds, yellows, and oranges are pigmented by carotenoids, acquired solely from the diet. Therefore, only individuals with superior carotenoid-foraging abilities should be colorful (Endler 1983; Kodric-Brown 1989; Hill 1992). However, carotenoids also have antioxidant and immunostimulant functions, suggesting an allocation trade-off where only the healthiest individuals can afford simultaneously to invest carotenoids in both pigmentation and self-maintenance (Lozano 1994; von Schantz et al. 1999). These are not mutually exclusive hypotheses, however, because healthy birds might also be the more capable individuals at accessing carotenoids when they are limited in the environment. Although a recent hypothesis suggests that carotenoid pigmentation might reflect a complex synthesis of different biochemical processes (Hill 2011; Hill and Johnson 2012), rather than access to carotenoids, it is most likely to be relevant for species that oxidize dietary carotenoids into pigmentary carotenoids (e.g., β-cryptoxanthin into red ketolated carotenoids; Hill and Johnson 2012). For birds that deposit dietary carotenoids unmodified into their integuments (e.g., lutein, zeaxanthin; the most common carotenoids used in pigmentation of yellow colors; McGraw 2006), the link between environmental carotenoid access and diet is predicted to be strong.

If dietary carotenoids are so important in determining plumage color, then it follows that their acquisition should be prioritized, especially during molt. There are several lines of evidence to suggest that birds might do this. First, within- and between-population differences in carotenoid-based plumage color have been attributed to dietary carotenoid access in great tits (*Parus major*) (Slagsvold and Lifjeld 1985; Isaksson 2009) and northern cardinals (*Cardinalis cardinalis*) (Linville and Breitwisch 1997). Even a species that deposits modified ketolated carotenoids into plumage, the house finch (*Carpodacus mexicanus*), shows a positive relationship between gut carotenoids content and carotenoid-based plumage color (Hill et al. 2002). Secondly, birds are capable of detecting small differences in nutrient concentration when feeding (e.g., lipid and sugar content; Schaefer et al. 2003) and of self-selecting a nutritionally balanced diet by combining foods that are individually imbalanced (Raubenheimer and Simpson 1997; Köhler et al. 2012). Thirdly, and perhaps most convincingly, choice tests with both captive and wild great tits have demonstrated that they prefer diets that are artificially carotenoid-enriched (Senar et al. 2010). Importantly, not all birds do so: Similar studies with house finches (Giraudeau et al. 2012) and garden warblers (*Sylvia borin*) (Catoni et al. 2011) have not detected these same carotenoid-foraging preferences. This could be because they either do not use carotenoids to color their plumage (garden warblers) or rely on more modified ketolated carotenoids to pigment their plumage (house finches). Therefore, to better link carotenoid-based signal function with the mechanism that maintains its honesty, a greater understanding of how carotenoid acquisition occurs in the wild, and how this is balanced against other nutritional requirements, is crucial (Olson and Owens 1998; Catoni et al. 2008).

To understand whether birds prioritize carotenoid acquisition, we explored whether colorful birds preferentially forage for carotenoids in the wild. During molt, birds deposit irretrievable carotenoids into their developing feathers. These colors will be present until their next molt—through winter to the next breeding season. For males to be at their most attractive to females, they must invest wisely. We studied the hihi (*Notiomystis cincta*), a species in which males possess a carotenoid-pigmented yellow shoulder badge important in territory acquisition and defense (Walker 2013). The pigments used to color their yellow feathers are predominantly the unmodified dietary carotenoids lutein and zeaxanthin (Ewen et al. 2006). Female hihi by comparison are less colorful. They do not share males’ carotenoid-pigmented plumage but instead are olive brown. During breeding, females may have elevated requirements for carotenoids (for yolk deposition), but during molt we predict that males should require more. This then should lead to them foraging preferentially for carotenoids compared with females during this period.

We first compared circulating carotenoid profiles of male and female hihi throughout the breeding season and during molt to determine how and when they differ. We next observed foraging behavior to ascertain if preferences exist. Female hihi are secretive during breeding/yolk deposition, so we limited our foraging observations to the molting period when they are as visible as males. To quantify whether any trade-offs in dietary nutrients exist, we measured the carotenoid, fat, and vitamin E content of fruit, invertebrates, and nectar occurring in the hihi diet. It is thought that carotenoids can be better utilized if consumed along with fat (Castenmiller and West 1998; Roodenburg et al. 2000; Surai 2002); therefore, we predicted that males will not only consume more carotenoids compared with females during molt but also more fat. Finally, we predicted that males would differ in their vitamin E intake compared with females. They may either have a greater vitamin E intake than females, in order to compensate the antioxidant function of carotenoids being lost to pigmentation, or a reduced vitamin E intake compared with females, to minimize competition with carotenoids during absorption.

## METHODS

### Study site and species

Hihi (*N. cincta*) are a sexually dimorphic and dichromatic passerine endemic to New Zealand. Males display yellow carotenoid-based (Ewen et al. 2006) shoulders and breast, a melanin-based black head, and structurally produced white ear tufts. Females are a less conspicuous olive brown all over, save from a white wing bar. Adult hihi eat nectar, fruits, and invertebrates, and the proportion of each food type in the diet appears to vary with season and population (Gravatt 1969; Gravatt 1971; Angehr 1984; Lovegrove 1985; Rasch 1985). Hihi molt once a year, immediately after the breeding season, at which time adults replace all of their feathers, and first-year birds replace their body feathers only (remiges and rectrices, grown in the nest, are retained until the next year’s molt). At this time, carotenoid pigments (lutein and zeaxanthin; Ewen et al. 2006) are deposited in the feather follicles. Adults typically molt between late December/early January and early April.

We studied the hihi on 220 ha Tiritiri Matangi Island (36°36′S, 174°53′E), which supports a population of ~180 color-ringed, individually identifiable, adult birds. The island was originally covered in coastal broadleaf forest, but following clearing for cultivation and grazing in the 19th century (Drey et al. 1982), and the subsequent replanting of native plants between 1983 and 1995 (Mitchell 1985), the island’s vegetation now comprises ~60% remnant and regenerating forest and ~40% grassland. Hihi inhabit the forested areas of the island, which contain diverse vegetation typical of the region. As part of a conservation management program, adults have access to supplementary sugar water feeding stations throughout the year (Armstrong and Ewen 2001) although data were not collected on feeder use during the study. Sugar water provides only carbohydrates; so ignoring feeder use potentially underestimated only this component of the diet. We do not believe that this had a significant bearing on our results as hihi use feeding stations minimally at this time of year (Walker LK, personal observation). There is also evidence that males use feeders to a greater extent than do females (Roper 2012) which, if accounted for, would only accentuate the observed difference of males having a greater carbohydrate intake (see Results).

### Plasma carotenoid profiles

Adult hihi were captured for blood sampling during 2 discrete periods: during the breeding season (September–December 2006) and during molt (January–March 2010). Samples from breeding birds were collected as part of a different study (Thorogood et al. 2011). Birds were captured at mist nets and feeding stations, and a blood sample was taken by brachial venipuncture. Blood samples were centrifuged within 3h to separate plasma, which was stored at −20 °C for subsequent analysis of plasma carotenoid concentration by high-performance liquid chromatography (HPLC), as described previously (Walker et al. 2013). A total of 67 samples from 45 individuals were collected during the breeding season, and 124 samples from 93 individuals during molt.

### Feeding observations

Feeding observations were collected from 18 January to 22 March 2010, when birds were molting. During the hours of peak bird activity (06:00–10:00 and 16:00–19:00), we walked the network of trails through forest patches and located and followed individual birds until they ate a natural food item. All forest patches were surveyed for approximately equivalent durations. The identity (color ring combination) and sex of the bird and the category of the first food item eaten (fruit/nectar/invertebrate) were recorded. Fruit and nectar were categorized according to species, and invertebrates according to order where possible. Observations of the same individual were separated by at least 1h, and only 1 food item was recorded per individual per sampling event. This sampling method has been used previously to estimate the proportions of different food categories in primate diets (Struhsaker 1975; Simmen and Sabatier 1996) and is appropriate when it is not practical to follow focal individuals for extended periods of time (e.g., Rothman et al. 2008; Felton et al. 2009). A total of 975 feeding observations from 233 different individual birds (males and females, including first-years) was made.

### Fruit sampling and nutritional analyses

Individual plants that hihi were observed taking fruit from were marked with flagging tape and returned to, usually later the same day but occasionally after a few days, for fruit sampling. Cabbage tree (*Cordyline australis*), hangehange (*Geniostoma ligustrifolium*), mahoe (*Melicytus ramiflorus*), mapou (*Myrsine australis*), five-finger (*Pseudopanax arboreus*), and *Coprosma robusta* each had 10 different individual plants flagged for sampling, and *Coprosma macrocarpa* and small-leaved *Coprosma* spp. (*Coprosma areolata* and *Coprosma rhamnoides*) each had 3 different individual plants flagged for sampling. Twenty ripe fruit were collected by hand from each flagged plant and within 2h were vacuum packed and stored at −20 °C. Due to time constraints, fruit were not sampled from kohekohe (*Dysoxylum spectabile*), kawakawa (*Macropiper excelsa*), puriri (*Vitex lucens*), and *Coprosma repens*. Hihi were only seen feeding on them very infrequently (collectively they account for just 4% and 6% of fruit in male and female diets, respectively), and this was probably due to inaccessibility rather than for nutritional reasons (kohekohe, kawakawa, and puriri are all too large for hihi to consume whole).

Half of the fruit samples collected were analyzed for individual and total carotenoid concentration and for individual and total vitamin E concentration. About 200–300mg fruit sample (excluding seeds) was saponified with ethanolic KOH in the presence of pyrogallol for 30min at 70 °C (Surai et al. 1996). After cooling, carotenoids were twice extracted by homogenization with hexane. Hexane extracts were pooled and evaporated under nitrogen and then redissolved in dichloromethane/methanol (1:1 v/v). Aliquots (10 μL) were injected into HPLC for analysis. Individual carotenoids were detected using a Spherisorb S30DS2 3μ C_18_ reverse-phase HPLC column (25cm × 4.6mm, Phenomex, Macclesfield, UK) with a mobile phase of acetonitrile/methanol (85:15 v/v) and acetonitrile/dichloromethane/methanol (70:20:10 v/v/v) in gradient elution and using detection at 445nm (Surai et al. 2001a, 2001b, 2001c). Total carotenoids were detected with a Spherisorb S5N2 DS2 5μ C_18_ reverse-phase HPLC column (25cm × 4.6mm, Phase Separations, Clwyd, UK) with a mobile phase of methanol/water (97:3 v/v) at a flow rate of 1.5mL/min. The HPLC was calibrated using carotenoid standards obtained from various sources. Vitamin E was determined using the same HPLC system (Shimadzu Liquid Chromatograph, LC-20AD, Japan Spectroscopic Co. Ltd with Fluorescence Spectrofluorometer) fitted with a Spherisorb ODS2 3μ C_18_ reverse-phase column (15cm × 4.6mm; Phase Separations) and using a mobile phase of methanol/water (97:3 v/v) at a flow rate of 1.05mL/min. The excitation and emission wavelengths were 295 and 330nm. A standard solution of α-tocopherol in methanol was used for instrumentation (HPLC) calibration. Carotenoid and vitamin E content was determined for all fruit species collected.

The remaining fruit samples were sent to Massey University’s Nutrition Laboratory (Palmerston North, New Zealand), where moisture, ash, crude protein, and fat content were determined according to the procedures of the Association of Official Analytical Chemists (AOAC 1990). In most cases, there was insufficient sample to perform repeat analyses, so samples from multiple individual plants were pooled to guarantee sufficient sample. In brief, samples were dried in a convection oven at 105 °C to determine moisture content (AOAC 930.15, 925.10). Total nitrogen was estimated using a Leco FP-528 combustion analyzer (AOAC 968.06), and crude protein was then calculated by multiplying total nitrogen by 6.25 (nitrogen to protein conversion factor). Fat content was determined by cold extraction using chloroform/methanol (AOAC 969.24). Finally, samples were placed in a 550 °C furnace for ~3h to obtain ash content (AOAC 942.05). Total carbohydrate content was determined as 100% minus percent protein, fat, ash, and moisture. There was sufficient sample to determine the nutritional content of cabbage tree (*C. australis*), hangehange (*G. ligustrifolium*), mahoe (*M. ramiflorus*), mapou (*M. australis*), five-finger (*P. arboreus*), *C. robusta*, and *C. macrocarpa*. There was insufficient sample remaining to determine the nutritional content of small-leaved *Coprosma* spp. (*C. areolata* and *C. rhamnoides*).

### Statistical analyses

Statistical analyses were carried out using R v. 2.15.1 (R Development Core Team 2011). First, we investigated whether plasma carotenoid concentration differed by sex and season. Because a large number of individuals had plasma carotenoid concentration measured multiple times, we fitted a linear mixed effects model using restricted maximum likelihood and included individual identity as a random effect. The response variable was plasma carotenoid concentration (Box–Cox transformed, to meet the assumptions of normality and homogeneity), and the explanatory variables were sex, season (breeding/molt), and an interaction between sex and season. To investigate whether plasma carotenoid concentration changed over the course of the molt, we fitted a second linear mixed effects model (using molt samples only) with sex, sampling date (centered Julian date), and a sex × sampling date interaction as explanatory variables.

Feeding observations during molt were used to estimate the proportional makeup of fruit, nectar, and invertebrates in the diets of males and females, and a chi-square test was used to test for sex differences in proportions of fruit, nectar, and invertebrates taken. The test was performed on a single contingency table because proportions of dietary intake are nonindependent of each other. The same approach was used to summarize the contribution of different fruit species to male and female diets. Because there were multiple observations from the same individuals, and there were a range of observations per individual (between 1 and 18 observations), it is possible that some individuals are “overrepresented” and may bias the results. To check for this, we used a resampling approach where we randomly sampled 1 observation per individual, used these sampled observations to estimate proportional makeup, and repeated this multiple times (*n* = 100). We then calculated the mean proportional makeup from this distribution and compared this with our original estimate (which used all observations at once).

Next, we recast feeding observations according to the carotenoid content of different fruit species. The 25th and 75th percentiles of fruit carotenoid concentration were calculated, and each sampled fruit species was then categorized according to these percentiles. Fruit species falling below the 25th percentile were categorized as low carotenoid content, fruit species falling between the 25th and 75th percentiles were categorized as medium carotenoid content, and fruit species falling above the 75th percentile were categorized as high carotenoid content (category assignment detailed in Table 1). The proportion of feeding observations that were on fruit of each of these carotenoid categories was then calculated for males and females, and a chi-square test was performed (on a single contingency table) to detect any sex differences in the proportions of high-, medium-, and low-carotenoid content fruits consumed (as above). The same approach was also taken for the vitamin E and fat content of fruits (category assignment detailed in Table 1).

**Table 1:** Macronutrient content (% wet weight) and carotenoid and vitamin E content (mean ± SE μg/g wet weight) of ripe fruit species fed on by hihi

Species	Macronutrient content (% wet weight)					Carotenoid content (μg/g wet weight)			Vitamin E content (μg/g wet weight)	
	Moisture	Ash	Protein	Fat	Carbohydrate	Lutein	Zeaxanthin	Total carotenoids	α-tocopherol	Total vitamin E
Cabbage tree	78.2	0.9	2.9	4.1 (M)	13.9	3.60±0.63	0.76±0.28	6.53±1.60 (M)	54.19±5.16	74.46±5.24 (M)
*Coprosma areolata* ^a^	—	—	—	—	—	7.73±3.23	0.00±0.00	8.53±2.42	53.01±33.54	66.40±38.76
*Coprosma macrocarpa*	73.3	0.4	0.9	2.2^b^ (L)	23.2	4.44	0.00	11.13 (M)	59.48	95.86 (H)
*Coprosma rhamnoides* ^a^	—	—	—	—	—	1.90	0.00	2.90	20.02	28.72
*Coprosma robusta*	72.6	0.6	1.4	2.5 (L)	22.9	4.25±0.69	0.16±0.07	19.63±5.06 (H)	47.55±8.43	61.75±9.80 (M)
Five-finger	72.2	1.2	2.0	4.4 (M)	20.2	8.28±1.30	0.00±0.00	10.77±1.42 (M)	44.60±6.39	65.03±9.02 (M)
Hangehange	72.0	1.2	2.4	5.9 (H)	18.5	38.08±3.74	0.00±0.00	81.65±5.45 (H)	33.45±6.62	159.64±7.79 (H)
Mahoe	75.3	1.1	3.6	5.8 (H)	14.2	0.55±0.31	0.08±0.04	1.36±0.77 (L)	40.30±2.70	72.88±5.35 (M)
Mapou	74.8	0.8	1.4	2.7 (L)	20.3	4.06±1.33	0.08±0.08	5.17±1.82 (M)	19.19±3.18	49.95±11.02 (L)

H, M, and L indicate which of high-, medium-, and low-nutritional content categories each fruit species falls into. See Supplementary Tables 2 and 3 for concentrations of additional forms of carotenoids and vitamin E.

^a^Insufficient sample to determine nutritional content.

^b^Value from unripe *C. macrocarpa*.

Finally, in order to visualize the nutrient content of sampled fruit species in a broader context, we used right-angled mixture triangles (RMTs; Raubenheimer 2011) to compare the nutritional content of sampled fruit species with published values of invertebrate orders that hihi potentially feed on (Diptera, Hemiptera, Lepidoptera; Ramos-Elorduy et al. 1997; Finke 2002; Banjo et al. 2006; Eeva et al. 2010; Raksakantong et al. 2010; O’Malley and Power 2012; Oonincx and Dierenfeld 2012; Finke 2013). Given the comparatively simple composition of nectar (mainly water and sugars; Nicolson et al. 2007), nectar is not represented in these plots. The RMT approach is well suited to field-based nutritional ecology studies where food items and dietary intake are described in terms of their proportional composition, rather than amounts (Raubenheimer 2011).

## RESULTS

### Plasma carotenoid profiles

**Figure 1:**
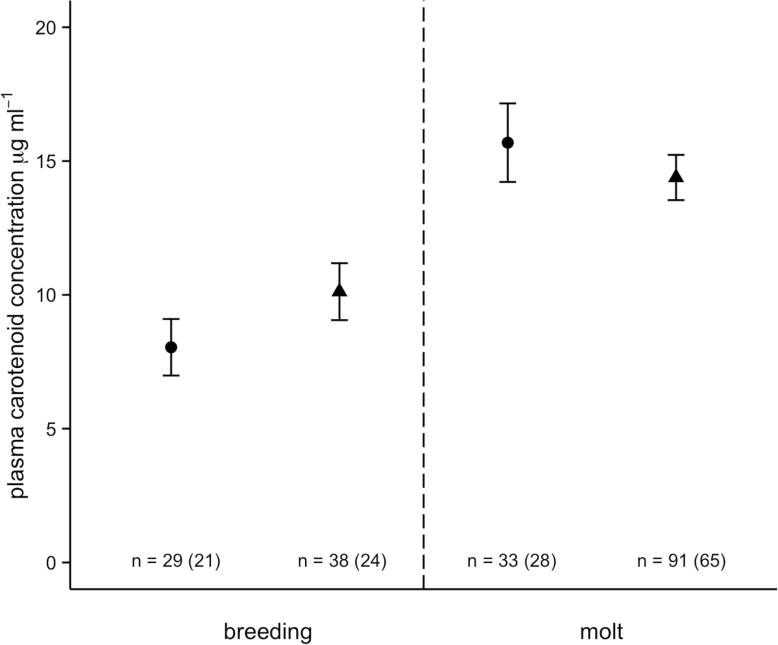
Plasma carotenoid concentrations (μg/mL) for female (circles) and male (triangles) hihi during breeding and molting (model estimates ± SE). Total number of samples per sex/season is indicated (with number of individual birds sampled in parentheses). Breeding season samples were collected September–December 2006, and molt samples were collected January–March 2010.

Plasma carotenoid concentration tended to be greater for males than females during the breeding season (mean model estimate, μg/mL ± standard error [SE]: 10.12±1.06 for males vs. 8.04±1.06 for females; Figure 1), and greater for females than males during molt (15.69±1.47 for females vs. 14.38±0.85 for males; Figure 1), although the interaction between sex and season was not significant (*t* = −1.24, *P* = 0.22). Overall, plasma carotenoid concentration was significantly greater during molt than during the breeding season (mean model estimate, μg/mL ± SE: 14.46±1.18 during molt vs. 9.03±0.92 during breeding season; *t* = 3.95, *P* = 0.0002; Figure 1). When the molt period was considered separately, there was a significant interaction between sex and date of measurement (*t* = 2.35, *P* = 0.03), indicating that males, but not females, showed a significant increase in plasma carotenoid concentration over the course of the molt.

### Feeding observations

**Figure 2:**
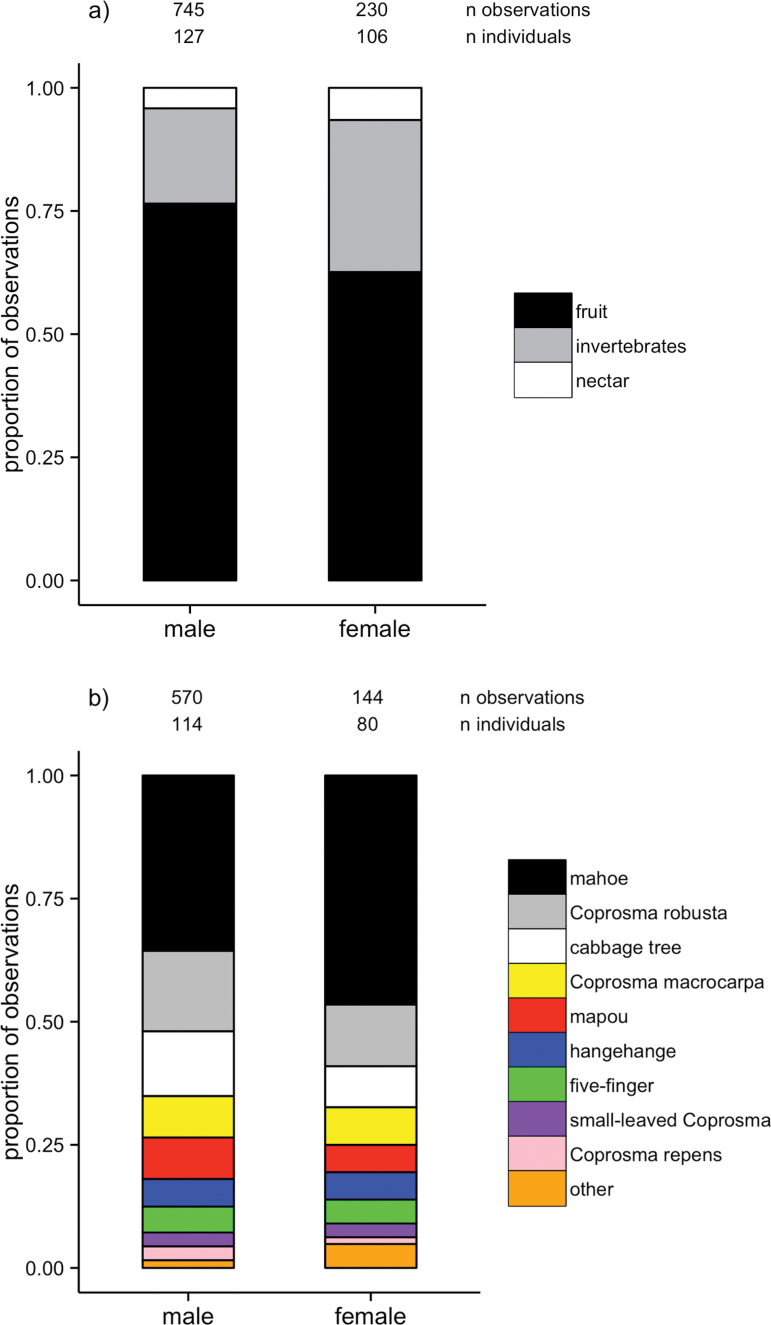
(a) The proportion of all feeding observations that were on fruit (black), invertebrates (gray), and nectar (white), and (b) the proportion of all fruit feeding observations that were on different fruit species, for males and females. The number of observations and number of different individuals are indicated. “Small-leaved *Coprosma*” refers to both *Coprosma areolata and Coprosma rhamnoides*. “Other” refers to large fruits that cannot be consumed whole (puriri, kohekohe, and kawakawa; see main text).

Our molt feeding observations revealed that males and females differed significantly in their proportional intake of fruit, nectar, and invertebrates (χ^2^ = 17.33, degrees of freedom [df] = 2, *P* < 0.001). Males had a greater proportion of fruit in their diet than did females (0.77 vs. 0.63; Figure 2a), a lesser proportion of invertebrates (0.19 vs. 0.31; Figure 2a), and a similar proportion of nectar (0.04 vs. 0.07; Figure 2a). The estimated proportional makeup did not differ markedly from this when a resampling approach was taken to account for some individuals being represented by multiple observations (Supplementary Table 1).

Males were observed feeding on 12 different species of fruit, and females on 11 different species of fruit (Figure 2b; Supplementary Table 1). Overall, there was a trend for the proportional intake of different fruit species to differ for males and females (χ^2^ = 20.02, df = 2, *P* = 0.05). This is largely driven by males feeding on a lesser proportion of mahoe than did females (0.36 vs. 0.47; Figure 2b; Supplementary Table 1). The proportion of all other fruit species in the diet, based on feeding observations, was similar for males and females (Figure 2b; Supplementary Table 1). The estimated proportional makeup of different fruit species did not differ markedly from these values when a resampling approach was taken (Supplementary Table 1).

### Nutritional content

**Figure 3:**
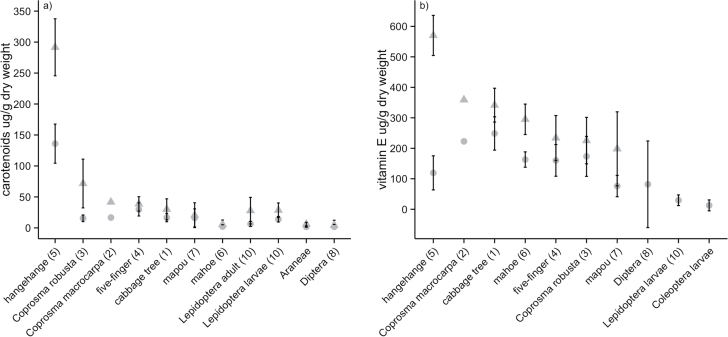
(a) Mean (± 95% confidence interval) total carotenoid (triangles) and lutein (circles) concentration (μg/g dry weight), and (b) mean (± 95% confidence interval) total vitamin E (triangles) and α-tocopherol (circles) concentration (μg/g dry weight) of different fruit species commonly fed on by hihi, and invertebrate orders potentially fed on by hihi (invertebrate data in (a) from Eeva et al. 2010; Finke 2013; invertebrate data in (b) from Finke 2002; Oonincx and Dierenfeld 2012; Finke 2013). Numbers in brackets correspond to fruit/invertebrate codes in legend of Figure 5.

The macronutrient (i.e., protein, fat, and carbohydrate), carotenoid, and vitamin E wet weight content of fruit species commonly fed on by hihi are summarized in Table 1. In most fruit species, lutein and α-tocopherol were the predominant forms of carotenoid and vitamin E, respectively (Table 1). Additional forms of carotenoids and vitamin E generally occurred at low concentrations (Supplementary Tables 2 and 3). Figure 3 compares the carotenoid and vitamin E concentrations (μg/g dry weight) of sampled fruit species with invertebrate orders potentially fed on by hihi. In general, invertebrates had a lower carotenoid and vitamin E content than hihi fruit (Figure 3).

**Figure 4:**
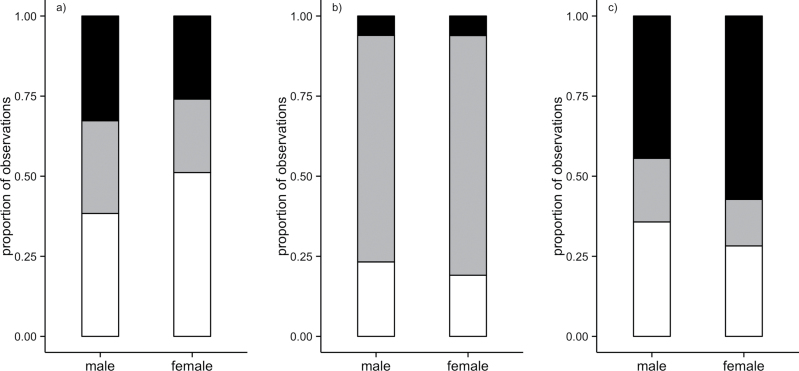
The proportion of male and female fruit feeding observations that were on fruit of low- (white), medium- (gray), and high- (black) carotenoid (a), vitamin E (b), and fat (c) content. *n* = 529 male observations (111 individuals), 131 female observations (72 individuals). Fruits in each low-, medium-, and high-content category are detailed in Table 1.

Recasting feeding observations according to the carotenoid content of fruits (see Table 1 for category assignment) revealed that males and females differed significantly in their proportional intake of high-, medium-, and low-carotenoid content fruit (χ^2^ = 6.67, df = 2, *P* = 0.04; Figure 4a). This is driven by males feeding on a lesser proportion of low-carotenoid content fruit than females (0.38 vs. 0.51; Figure 4a). Males and females did not differ in their consumption of fruit based on vitamin E content (χ^2^ = 1.22, df = 2, *P* = 0.54; Figure 4b) but did differ in their consumption of fruit based on fat content (χ^2^ = 6.55, df = 2, *P* = 0.04; Figure 4c). The latter result was driven by males feeding on a lesser proportion of high-fat content fruit than females (0.44 vs. 0.57; Figure 4c). Both of these relationships (carotenoid and fat) appear to be driven by the relative preference of females for mahoe and a preference of males for fruit other than mahoe. This fruit is both low in carotenoid content and high in fat content (Table 1).

**Figure 5:**
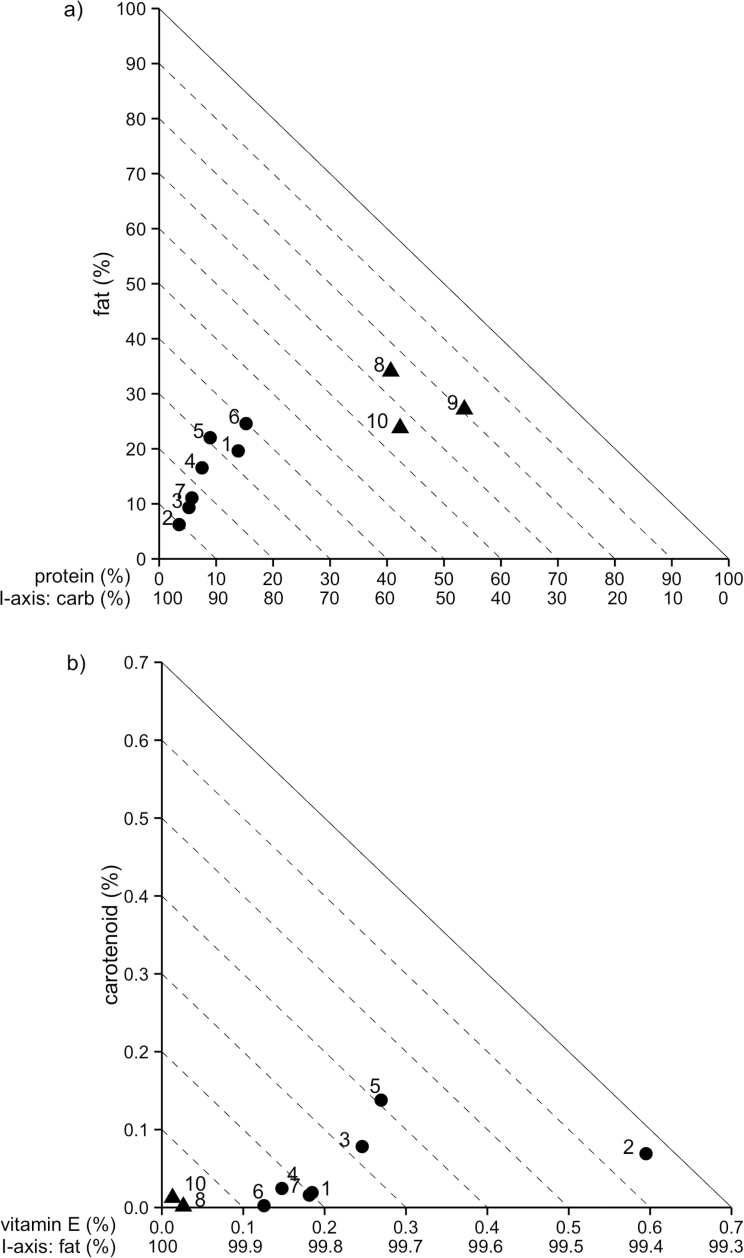
RMTs showing (a) the proportional protein, fat, and carbohydrate composition, and (b) the proportional carotenoid, vitamin E, and fat composition of fruit species fed on by hihi (circles), and invertebrate orders potentially fed on by hihi (triangles). In a 3-component RMT plot, 2 components are represented as standard *x* and *y* axes (protein and fat in (a), carotenoid and vitamin E in (b)), and the third component (the “implicit” axis, carbohydrate in (a) and fat in (b)) varies inversely with the distance from the origin. The value of the implicit axis is read by following the dashed lines from the point of interest to the *x* axis and reading the value of the *I* axis legend (Raubenheimer 2011). Percentages are based on dry mass in grams. Fruit: 1 = cabbage tree, 2 = *Coprosma macrocarpa*, 3 = *Coprosma robusta*, 4 = five-finger, 5 = hangehange, 6 = mahoe, 7 = mapou. Invertebrates: 8 = Diptera, 9 = Hemiptera, 10 = Lepidoptera. Invertebrate data are averaged from references cited in text.

An RMT plot of proportional protein, fat, and carbohydrate composition revealed hihi fruit to be of lower protein content, lower fat content, and higher carbohydrate content than potential invertebrate food items (Figure 5a). An RMT plot of proportional carotenoid, vitamin E, and fat composition reveals that fruit with a proportionally high carotenoid content tend to also have a proportionally high vitamin E content and a proportionally low fat content (Figure 5b). The positive relationship between proportional carotenoid and vitamin E content is, however, not significant (Kendall’s rank correlation, *r* = 0.07, *P* = 0.62), suggesting that carotenoid and vitamin E intake can vary independently of each other. These plots also emphasize that mahoe, of all fruit species measured, is most similar in composition to invertebrates, having the highest protein and fat content, and lowest carotenoid content (Figure 5).

## DISCUSSION

We compared the nutrient content of food items taken by male and female hihi to investigate whether males, the more colorful sex, target carotenoid-rich food during molt. Insodoing, we assumed that each sex had equal access to each type of food and that one sex did not competitively exclude the other, but this assumption remains to be tested in future work.

### Males target carotenoid-rich food during molt

We found that male hihi appear to target carotenoid-rich foods during molt. Firstly, males ate proportionally more fruit than females, and these fruit had a higher carotenoid content than other components of the diet. Secondly, within the fruit component of the diet, males selected the most carotenoid-rich fruits. In contrast, females ate proportionally more invertebrates than males, and the fruit they preferred was the most invertebrate-like in its composition. This is the first study of which we are aware to show a dietary carotenoid preference in the wild during molt by individuals with carotenoid-colored plumage.

Despite this carotenoid preference, male hihi did not have higher plasma carotenoid concentrations than females over the molt period. Although higher levels of circulating carotenoids might be expected in the sex with greater carotenoid demand (Hill 1995a, 1995b; Figuerola and Gutierrez 1998; Negro et al. 1998, 2001; McGraw et al. 2003), it is equally possible that lower circulating carotenoid levels simply reflect the fact carotenoids are being used to pigment new feathers and so are not free to circulate in the blood (Peters et al. 2007; Biard et al. 2009; Alonso-Alvarez and Galvan 2011). Consistent with this possibility is the observation that the yellow feathers of hihi are among the first feathers to be replaced in the molt sequence (Walker LK, personal observation). Perhaps, the increase in circulating carotenoids seen in male hihi through the course of the molt is a consequence of a reduced requirement for carotenoid deposition into feathers during the latter stages of molt.

Our findings are consistent with recent studies suggesting that birds are able to detect carotenoid presence in artificially manipulated food and adjust their consumption in response (Senar et al. 2010; Catoni et al. 2011). Great tits maximize their carotenoid intake by preferentially choosing carotenoid-enriched mealworms (Senar et al. 2010), while garden warblers are able to detect carotenoid presence and maintain a consistent (although not necessarily maximal) carotenoid intake (Catoni et al. 2011). In contrast, house finches do not display an ability to detect or maximize dietary carotenoids, at least based on a specific olfactory cue (Giraudeau et al. 2012). We suggest that the nature of the carotenoids used for pigmentation may explain the discrepancies between these studies. Species that pigment their feathers with unmodified dietary carotenoids are those most likely to maximize their carotenoid intake. Species that use endogenously modified carotenoids are less likely to maximize carotenoid intake because of the relatively greater importance of physiological processes compared with dietary access in determining color production (Hill and Johnson 2012). Finally, species without carotenoid pigmentation only require dietary carotenoids for the remaining properties, such as antioxidant function, egg production, and vision (roles that can often be fulfilled by other compounds; Svensson and Wong 2011), and are therefore the least likely to maximize intake.

As well as predicting male carotenoid intake to be greater than for females, we also predicted that intake of vitamin E would be different for males and females. However, our results instead suggest that vitamin E intake was similar for both sexes. At first sight, our finding that carotenoid and vitamin E intakes are not simultaneously maximized is not consistent with the influential prediction that carotenoid-based signals indicate the availability of other antioxidant resources such as vitamin E (Hartley and Kennedy 2004). However, we have only considered 1 alternative antioxidant, and the intake of other relevant resources, such as vitamin C and antioxidant enzymes, may still be tied to carotenoid intake in a manner consistent with Hartley and Kennedy’s (2004) hypothesis.

### Females target protein- and fat-rich food during molt

We also predicted that males would be more likely to target fat-rich food than females because this could aid the absorption of carotenoids. Instead, we found the reverse pattern. Perhaps it is not so surprising that the greater intake of carotenoids by males is not accompanied by a corresponding increase in fat intake. Fats have a vast range of functions besides aiding carotenoid absorption; for example, they are a source of energy, are important cell membrane components, and serve as nerve “insulators” (Cheeke and Dierenfeld 2010). Rather, the driver behind the differing male and female requirements for fat may be unrelated to their interaction with carotenoids and may instead be due to a contrast in the nutritional demands of the 2 sexes. Although males favored a high carotenoid diet during molt, females targeted food with a high fat and/or protein content (Maklakov et al. 2008; Morehouse et al. 2010). Perhaps, the protein and fat intake by females is greater during molt because they have a greater need to compensate the nutritional demands of breeding, such as those imposed by nourishing eggs with protein and fat.

Our observations for patterns of carotenoid use by females are harder to interpret because females become relatively secretive during the breeding season and this makes it difficult to collect comparable foraging data for the 2 sexes. For this reason, our comparison between the sexes during the breeding season is confined to measures of carotenoid concentration in the plasma. We would expect that females have a higher carotenoid requirement than males at this time because they are depositing carotenoids into egg yolk. This might explain why plasma carotenoid concentrations are lower for females during the breeding season than during the molt. Alternatively, females may consume fewer carotenoid-rich foods during the breeding season because their diet is dictated by the needs of their young or because there are fewer carotenoid-rich fruit available then. Both sexes have higher plasma carotenoid concentrations during the molt, which is consistent with the latter possibility. Similar patterns have also been reported in a Mediterranean population of great tits (*P. major*; del Val et al. 2013), where availability of carotenoids may drive the seasonal variation in circulating carotenoids.

We are aware of only 1 hihi study that compares male and female proportional intake throughout the year (Angehr 1984). This suggests that males also eat more fruit than females when they are not molting, implying that carotenoid intake is greater for males year-round. Interestingly, the carotenoid preference in great tits was also demonstrated outside of molt (Senar et al. 2010). Perhaps, in those cases male physiology is primed to maintain a high carotenoid content throughout the year. Within species, the more colorful sex may require a year-round higher carotenoid intake than the less colorful sex. Alternatively, there might not be a year-round higher requirement for carotenoids by males, but sustained foraging for carotenoid-rich foods might provide the information necessary for obtaining the required levels during molting. Which, if either, of these explanations apply would be an interesting direction for future research.

In conclusion, our results provide support for the hypothesis that colorful male hihi target carotenoid-rich foods during molt. However, we do not find any evidence that other components of the diet are regulated in a manner consistent with maximizing carotenoid availability. Further work is needed to establish whether such patterns are maintained outside of molt and to clarify the role of female foraging behavior in shaping these relationships.

## SUPPLEMENTARY MATERIAL

Supplementary material can be found at http://www.beheco.oxfordjournals.org/.

## FUNDING

This work was supported by the Natural Environment Research Council (PFAG/030 to L.K.W.) and the Leverhulme Trust (F/00 390/E to J.G.E.).

## Supplementary Material

Supplementary Data
