# Supplementary material for: Foraging for carotenoids: do colorful male hihi target carotenoid-rich foods in the wild?
Source: Behav Ecol. 2014 May 12;25(5):1048–57. doi: 10.1093/beheco/aru076 (PMC4160110; doi:10.1093/beheco/aru076)
Supplement: Supplementary Data [file supp_25_5_1048__index.html]

Foraging for carotenoids: do colorful male hihi target carotenoid-rich foods in the wild? — Foraging for carotenoids: do colorful male hihi target carotenoid-rich foods in the wild? — Supplementary Data 

# Foraging for carotenoids: do colorful male hihi target carotenoid-rich foods in the wild?

## Supplementary Data

Data files

**Files in this Data Supplement:**

- Supplementary Data - Supplementary Data
