# Supplementary material for: Foraging for carotenoids: do colorful male hihi target carotenoid-rich foods in the wild?
Source: Behav Ecol. 2014 May 12;25(5):1048–57. doi: 10.1093/beheco/aru076 (PMC4160110; doi:10.1093/beheco/aru076)
Supplement: Supplementary Data [file supp_aru076_supplementary_tables_260114.doc]

**Table S1:** Observed and mean re-sampled proportions of a) different food categories, and b) different fruit species occurring in the diets of males and females. Significant differences are highlighted in bold. Mean re-sampled proportion refers to the mean proportion following re-sampling (where one observation per individual was randomly sampled to generate an estimate of proportional make-up, and this was repeated 100 times).

|  | observed proportion | | mean re-sampled proportion (95% CI) | |
| --- | --- | --- | --- | --- |
| food item | males | females | males | females |
|  |  |  |  |  |
| a) food category |  |  |  |  |
| fruit | 0.77 | 0.63 | 0.7548 (0.7493 – 0.7603) | 0.6286 (0.6245 – 0.6326) |
| invertebrates | 0.19 | 0.31 | 0.1566 (0.1516 – 0.1616) | 0.2888 (0.2850 – 0.2926) |
| nectar | 0.04 | 0.07 | 0.0886 (0.0860 – 0.0912) | 0.0826 (0.0801 – 0.0852) |
|  |  |  |  |  |
| b) fruit species^a^ |  |  |  |  |
| cabbage tree | 0.13 | 0.08 | 0.1339 (0.1290 – 0.1389) | 0.0725 (0.0693 – 0.0757) |
| *C. macrocarpa* | 0.08 | 0.08 | 0.1144 (0.1112 – 0.1176) | 0.0709 (0.0681 – 0.0736) |
| *C. repens* | 0.03 | 0.01 | 0.0316 (0.0290 – 0.0342) | ?? (?? – ??) |
| *C. robusta* | 0.16 | 0.13 | 0.1626 (0.1581 – 0.1672) | 0.1369 (0.1327 – 0.1411) |
| five-finger | 0.05 | 0.05 | 0.0592 (0.0558 – 0.0626) | 0.0520 (0.0499 – 0.0541) |
| hangehange | 0.06 | 0.06 | 0.0525 (0.0498 – 0.0552) | 0.0425 (0.0394 – 0.0456) |
| kawakawa | 0.005 | 0.03 | 0.0028 (0.0019 – 0.0037) | 0.0503 (0.0485 – 0.0520) |
| kohekohe | 0.005 | 0.01 | 0.0042 (0.0031 – 0.0054) | 0.0169 (0.0157 – 0.0181) |
| mahoe | 0.36 | 0.47 | 0.3275 (0.3212 – 0.3337) | 0.4400 (0.4338 – 0.4462) |
| mapou | 0.08 | 0.06 | 0.0734 (0.0695 – 0.0773) | 0.0564 (0.0535 – 0.0592) |
| puriri | 0.005 | 0.00 | 0.0053 (0.0041 – 0.0064) | NA |
| small-leaved *Coprosma* spp. | 0.03 | 0.03 | 0.0325 (0.0307 – 0.0344) | 0.0368 (0.0350 – 0.0385) |
|  |  |  |  |  |

^a^ Fruit proportions are expressed as proportion of all fruit species

**Table S2:** Individual and total carotenoid content (mean ± SE μg/g wet weight) of ripe fruit species fed on by hihi. H, M, and L indicate which of high-, medium- and low-carotenoid content categories each fruit species falls into.

|  |  | μg/g wet weight | | | | | | | | |
| --- | --- | --- | --- | --- | --- | --- | --- | --- | --- | --- |
| species | n | lutein | zeaxanthin | *cis*-lutein | anhydrolutein | echinenone | β-crypotoxanthin | α-carotene | β-carotene | total carotenoids |
|  |  |  |  |  |  |  |  |  |  |  |
| cabbage tree | 9 | 3.60 ± 0.63 | 0.76 ± 0.28 | 0.09 ± 0.06 | 0.00 ± 0.00 | 0.00 ± 0.00 | 0.10 ± 0.10 | 0.56 ± 0.56 | 1.14 ± 0.47 | 6.53 ± 1.60 (M) |
| *C. areolata* | 2 | 7.73 ± 3.23 | 0.00 ± 0.00 | 0.00 ± 0.00 | 0.23 ± 0.23 | 0.00 ± 0.00 | 0.00 ± 0.00 | 0.00 ± 0.00 | 0.58 ± 0.58 | 8.53 ± 2.42 |
| *C. macrocarpa* | 1 | 4.44 | 0.00 | 0 | 0 | 0 | 0 | 3.5 | 2.46 | 11.13 (M) |
| *C. rhamnoides* | 1 | 1.90 | 0.00 | 0 | 0 | 0 | 0 | 0.54 | 0.46 | 2.90 |
| *C. robusta* | 16 | 4.25 ± 0.69 | 0.16 ± 0.07 | 0.17 ± 0.11 | 0.07 ± 0.04 | 0.16 ± 0.11 | 1.94 ± 0.82 | 8.46 ± 2.67 | 3.85 ± 1.05 | 19.63 ± 5.06 (H) |
| five-finger | 10 | 8.28 ± 1.30 | 0.00 ± 0.00 | 0.19 ± 0.07 | 0.05 ± 0.05 | 0.00 ± 0.00 | 0.03 ± 0.03 | 0.14 ± 0.14 | 1.55 ± 0.36 | 10.77 ± 1.42 (M) |
| hangehange | 8 | 38.08 ± 3.74 | 0.00 ± 0.00 | 1.73 ± 0.57 | 0.00 ± 0.00 | 7.76 ± 1.46 | 0.48 ± 0.15 | 0.00 ± 0.00 | 27.82 ± 3.21 | 81.65 ± 5.45 (H) |
| mahoe | 9 | 0.55 ± 0.31 | 0.08 ± 0.04 | 0.00 ± 0.00 | 0.00 ± 0.00 | 0.00 ± 0.00 | 0.00 ± 0.00 | 0.00 ± 0.00 | 0.72 ± 0.41 | 1.36 ± 0.77 (L) |
| mapou | 5 | 4.06 ± 1.33 | 0.08 ± 0.08 | 0.09 ± 0.06 | 0.00 ± 0.00 | 0.00 ± 0.00 | 0.00 ± 0.00 | 0.00 ± 0.00 | 0.94 ± 0.44 | 5.17 ± 1.82 (M) |
|  |  |  |  |  |  |  |  |  |  |  |

**Table S3:** Individual and total vitamin E content (mean ± SE μg/g wet weight) of ripe fruit species fed on by hihi. H, M, and L indicate which of high-, medium- and low-vitamin E content categories each fruit species falls into.

|  |  | | μg/g wet weight | | | | | |
| --- | --- | --- | --- | --- | --- | --- | --- | --- |
| species | | n | α-tocopherol | δ-tocopherol | γ-tocopherol | α-tocotrienol | γ-tocotrienol | total vitamin E |
|  | |  |  |  |  |  |  |  |
| cabbage tree | | 9 | 54.19 ± 5.16 | 0.03 ± 0.02 | 19.33 ± 2.40 | 0.81 ± 0.79 | 0.10 ± 0.06 | 74.46 ± 5.24 (M) |
| *C. areolata* | | 2 | 53.01 ± 33.54 | 0.49 ± 0.08 | 12.87 ± 5.33 | 0.04 ± 0.04 | 0 | 66.40 ± 38.76 |
| *C. macrocarpa* | | 1 | 59.48 | 2.37 | 33.59 | 0.22 | 0.19 | 95.86 (H) |
| *C. rhamnoides* | | 1 | 20.02 | 0.16 | 8.48 | 0.06 | 0 | 28.72 |
| *C. robusta* | | 16 | 47.55 ± 8.43 | 0.73 ± 0.34 | 13.15 ± 3.74 | 0.17 ± 0.07 | 0.15 ± 0.07 | 61.75 ± 9.80 (M) |
| five-finger | | 10 | 44.60 ± 6.39 | 3.70 ± 0.79 | 12.61 ± 2.23 | 3.62 ± 1.45 | 0.50 ± 0.16 | 65.03 ± 9.02 (M) |
| hangehange | | 8 | 33.45 ± 6.62 | 4.64 ± 1.08 | 117.8 ± 9.06 | 2.08 ± 1.14 | 1.68 ± 0.36 | 159.64 ± 7.79 (H) |
| mahoe | | 9 | 40.30 ± 2.70 | 0.34 ± 0.11 | 28.11 ± 4.13 | 3.30 ± 0.57 | 0.82 ± 0.25 | 72.88 ± 5.35 (M) |
| mapou | | 5 | 19.19 ± 3.18 | 1.35 ± 0.76 | 28.28 ± 11.40 | 1.09 ± 0.88 | 0.04 ± 0.04 | 49.95 ± 11.02 (L) |
|  | |  |  |  |  |  |  |  |
